# Shifting the Sun: Solar Spectral Conversion and Extrinsic Sensitization in Natural and Artificial Photosynthesis

**DOI:** 10.1002/advs.201500218

**Published:** 2015-12-02

**Authors:** Lothar Wondraczek, Esa Tyystjärvi, Jorge Méndez‐Ramos, Frank A. Müller, Qinyuan Zhang

**Affiliations:** ^1^ Otto Schott Institute of Materials ResearchUniversity of JenaJena07743Germany; ^2^ Centre for Energy and Environmental Chemistry (CEEC)University of JenaJena07743Germany; ^3^ Department of Biochemistry and Food ChemistryUniversity of Turku20014TurkuFinland; ^4^ Department of PhysicsUniversity La Laguna38206La LagunaTenerifeSpain; ^5^ State Key Laboratory of Luminescent Materials and DevicesInstitute of Optical Communication MaterialsSouth China University of TechnologyGuangzhou510640P.R. China

**Keywords:** artificial photosynthesis, photosynthesis, solar spectral conversion, solar‐to‐fuel, water splitting

## Abstract

Solar energy harvesting is largely limited by the spectral sensitivity of the employed energy conversion system, where usually large parts of the solar spectrum do not contribute to the harvesting scheme, and where, of the contributing fraction, the full potential of each photon is not efficiently used in the generation of electrical or chemical energy. Extrinsic sensitization through photoluminescent spectral conversion has been proposed as a route to at least partially overcome this problem. Here, we discuss this approach in the emerging context of photochemical energy harvesting and storage through natural or artificial photosynthesis. Clearly contrary to application in photovoltaic energy conversion, implementation of solar spectral conversion for extrinsic sensitization of a photosynthetic machinery is very straightforward, and—when compared to intrinsic sensitization—less‐strict limitations with regard to quantum coherence are seen. We now argue the ways in which extrinsic sensitization through photoluminescent spectral converters will—and will not—play its role in the area of ultra‐efficient photosynthesis, and also illustrate how such extrinsic sensitization requires dedicated selection of specific conversion schemes and design strategies on system scale.

## 1 Introduction

**Figure 1:**
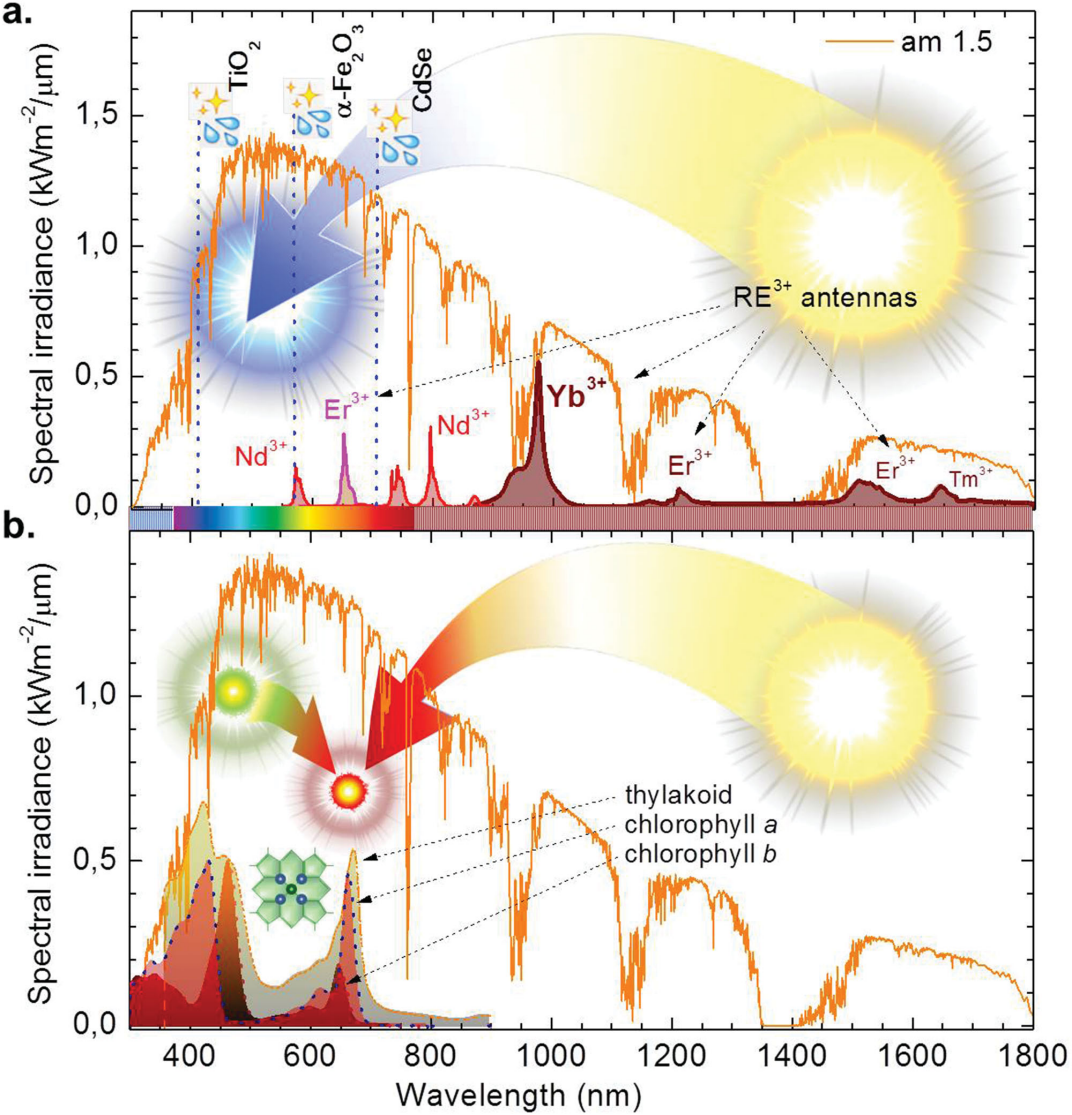
Solar spectral irradiance and spectral sensitivity of photochemical harvesting processes. a) Selected applications of artificial photosynthesis are depicted through the position of the optical bandgap of typical photocatalytic agents, TiO_2_ (rutile), α‐Fe_2_O_3_ and CdSe. For comparison, we also show the characteristic absorption bands of some rare‐earth antenna which may be employed in up‐conversion phosphors. b) The same spectrum, here in comparison to absorption spectra of chlorophylls *a* and *b* in methanol solution, and in pumpkin thylakoids. Pigment data of (b) was adopted from Frigaard et al.^138^ thylakoid data from Antal et al.^93^ The solar spectrum and exemplary data shown in (a) have been adopted from Refs. 88, 92.

In most techniques of solar energy harvesting, only a fraction of the incoming photon energy can be put to use in the targeted energy conversion process. The reasons for this are manifold and arise at different levels of the system. For example, regardless of the intrinsic quantum efficiency, the overall absorption cross‐section is often limited by surface reflectivity on module scale, or, in some cases, the optical transparency of the photon converter, subject to the considered spectral regime of the incoming light. While the former has motivated the use of thin coatings to adjust reflection, re‐emission, and emissivity, the latter requires dedicated material adjustments which may range from an enhanced path‐length of light interaction through tailored scattering properties, to chemical adjustments on materials scale, or to the introduction of photonic antenna and resonators. Another, even more prominent issue is the spectral selectivity of the employed conversion process, where the full spectral bandwidth of solar irradiation can usually not be used within a single harvesting and conversion scheme and/or where a photochemical or photoelectronic reaction cannot make use of the photon energy which is in excess of the respective band‐gap of the system. In particular in photovoltaic energy conversion (PV), these two aspects are well‐known as the Shokley–Queisser limit.^1^ That is, not all photons which form the solar spectrum participate in the conversion process, and of those which participate, their full energetic potential is not used. On the contrary, high‐energy ultraviolet photons may even have negative influence on the conversion scheme due to their causing recombination reactions, photo‐bleaching, or other effects of degradation, and are therefore often blocked from the converter cell. This problem of spectral mismatch is depicted in **Figure** **1**. In PV, two approaches are followed to address these issues, i.e., the design of multi‐junction devices which combine two or more semiconductors or photoelectric chromophores with adjusted spectral sensitivity to cover the largest part of the incoming spectrum,^2^ and luminescent converter materials which absorb specific parts of the spectrum and re‐emit in the sensitive region of the PV cell.^3^, ^4^, ^5^ The latter approach has been termed *spectral conversion*, with its presently most prominent variant, *spectral up‐conversion*, in which photons of sufficient energy to pass the electronic bandgap of the solar cell are generated from the low‐energy near infrared (NIR) tail of the solar spectrum. Here, we refer to this process as extrinsic sensitization, where the incoming spectrum is modified to improve its match with the acceptor material. In analogy, intrinsic sensitization refers to (chemical) modification of the acceptor to adjust or broaden its spectral selectivity.

In contrast to intrinsic sensitization such as occurs in the archetype dye‐sensitized solar cell,^6^ however, the actual benefit of extrinsic sensitization of solar cells through photoluminescent spectral converters remains unclear, as it imposes a significant increase in system complexity. Besides the simple notion of cost, this is accompanied by a variety of physical issues which have as yet prevented any real‐world relevance for PV applications.

Photochemical reactions and, in particular, natural or artificial photosynthesis present a somewhat different situation. Here, pigments (or dyes) with specific absorption properties, or large‐bandgap semiconductors are used to harvest incoming photons of a certain spectral range to generate electrons for transfer to a photosynthetic machinery. Adjustments of spectral sensitivity through extrinsic spectral conversion appears more straightforward in these cases because of less‐strict boundary conditions—compared to PV—with respect to the overall spectral properties of the converter material and its integration on module scale.^7^ Spectral adjustment through luminescent photoconverters has thus been demonstrated as an emerging approach for enhancing the photosynthetic activity of microalgae^7^ as well as that of higher plants.^8^, ^9^ Here, we present a concise progress report and a prospective outlook on this topic.

## 2 Photosynthetic Energy Harvesting

The evolution of photosynthesis began in water, and aquatic photosynthesis is still responsible for more than half of the global primary biomass production. Algae, cyanobacteria and submerged aquatic plants rely on inorganic carbon in the forms of bicarbonate and carbon dioxide. Thereby, virtually all aquatic photosynthesizers use metabolic energy to pump this limiting raw material into the cells.^10^ Carbon (dioxide) concentrating mechanisms are needed because photosynthesis rapidly consumes inorganic carbon from the vicinity of the cell. Therefore, carbon storage mechanisms which operate during low photosynthetic activity are an important feature. In spite of the slow raw material diffusion (which necessitates these carbon concentrating mechanisms), aquatic photosynthesis can be highly productive^11^, ^12^ because non‐photosynthetic supporting structures like roots and stem are not necessary in water. For example, in dilute suspension, some unicellular algae and cyanobacteria divide several times per day, which can lead to extreme productivity in terms of biomass (and, as a sometimes consequence, lipid) turnover.^13^ This has led to very strong interest in exploiting aquatic photosynthesis for CO_2_ storage, and also for the production of biofuel, fine chemicals, food or, e.g., cosmetic products.^11^, ^12^, ^14^, ^15^ However, the productivity of aquatic photo­synthesis decreases rapidly when the cell density increases, mostly because of mutual shading. Therefore, the use of aquatic microorganisms for the production of biofuels like biodiesel or biohydrogen^16^, ^17^, ^18^ requires concerted efforts of biotechnology and engineering of growth facilities^19^ for better gas exchange^20^ and, in particular, light management (e.g.,^7^, ^21^).

**Figure 2:**
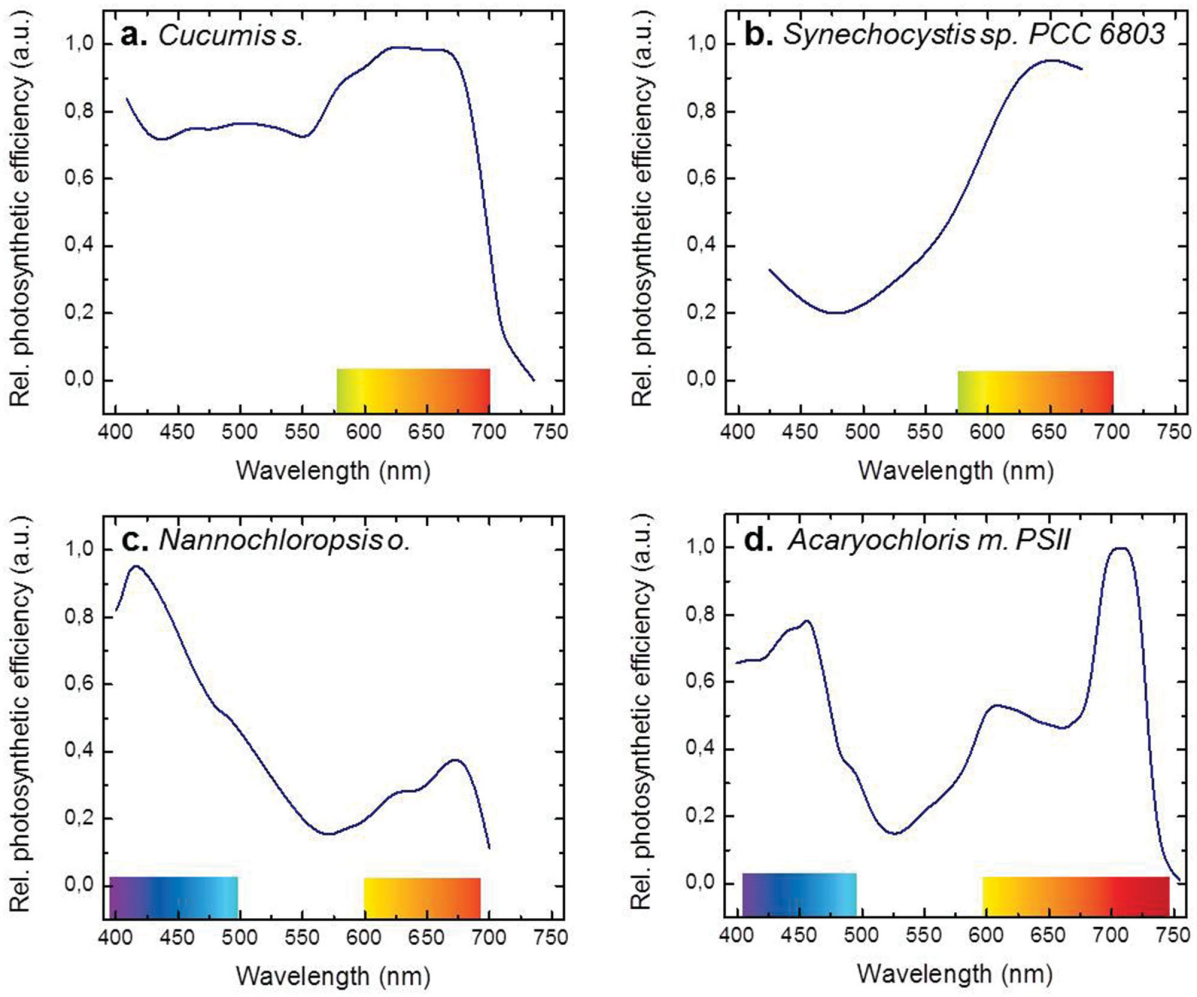
Action spectra of photosynthesis in a) cucumber leaves, b) the cyanobacterium *Synechocystis* sp. PCC 6803, and c) the chlorophyll‐*a*‐containing microalga *Nannochloropsis oculata*. d) Photochemical action spectrum of photosystem II in the chlorophyll‐*d*‐containing cyanobacterium *Acaryochloris marina*. The schematic data are redrawn from Refs. 23, 24, 25, 26, 27, 28.

Oxygenic photosynthesis uses wavelengths of visible light, but the actual efficiency of each wavelength is determined by the light‐harvesting machinery of photosynthesis.^22^ The wavelength‐regime above 700 nm is typically not efficiently usable in oxygenic photosynthesis, with the maximum absorption cross section of photosystem II (PSII) at 680 nm, and that of photosystem I (PSI) at 700 nm (here, the position of the absorbance peak of the photosynthetic reaction center can be understood as the equivalent to the bandgap of the semiconductor in PV). Ultraviolet radiation, in turn, may be efficiently absorbed by non‐photosynthetic components of the cells and does, hence, not very efficiently participate in photosynthesis. Thus, the photosynthetically most active range of wavelengths lies between about 400 to 700 nm (see also Figure 1b). In a first consideration, the match between solar spectral irradiance and the spectral harvesting coverage of the main pigments is poor, as chlorophyll *a*, the ubiquitous pigment found in (nearly) all oxygenic photosynthetic organisms, absorbs only blue and red light and is virtually transparent in green and yellow. The absorption spectrum of thylakoid membranes (which comprise the pigments) shows that the gap in the absorbance of chlorophyll *a* is partially covered by auxiliary pigments (such as carotenoids in all organisms; chlorophyll *b* in plants; chlorophylls *c, d*, or *f* and phycobilins in various cyanobacteria and algae), and also by changes in the absorption properties due to interaction of the pigments with the protein matrix. However, even in the light of these factors, the action spectrum of photosynthesis is relatively flat in typical leaves of land plants (**Figure** **2**a,^23^), revealing that the efficiency of each wavelength is greatly affected by the number of pigment molecules per unit area or volume. In fact, even green light has been shown to be highly efficient in plant photosynthesis,^24^ probably because green light penetrates deep into the leaf tissue.

The evolutionary strategy in which the cell piles‐up a large number of pigment molecules is not viable in aquatic photosynthesis because the slow diffusion of gases, especially CO_2_, limits the thickness of photosynthetic tissue, favoring small, thin, unicellular or filamentous organisms. Consequently, the action spectrum of photosynthesis obtained from a layer of aquatic organisms closely resembles the absorption spectrum of the ensemble of photosynthetic pigments of these organisms. For example, orange and red light (600–700 nm) are 2–5 times as efficient in causing photosynthesis in a suspension of cyanobacteria (*Synechocystis* sp. PCC 6803) than other wavelength ranges (Figure 2b,^25^). Similarly, the industrially important chromalveolate microalga *Nannochloropsis oculata* uses most efficiently the violet and blue spectral ranges (Figure 2c,^26^).

Chlorophylls are lipophilic pigments bound to integral membrane proteins via coordination bonds of the central magnesium atom. All variants of chlorophylls absorb blue light. In the red region, chlorophylls *a, b* and *c* are practically transparent above 700 nm, whereas organisms containing chlorophylls *d* or *f* have significant absorption up to ≈750 nm.^27^ For example, in the chlorophyll‐*d*‐containing cyanobacterium *Acaryochloris marina*, photosynthesis appears to be driven by near‐infrared light within the spectral range 700 to 750 nm, at a rate which is proportional to the absorbance (Figure 2d,^28^). Chlorophyll*‐d*‐containing organisms have consequently been studied for possibilities to extend the wavelength range of photosynthesis towards the near‐infrared.^29^ As another example, the antenna pigments behind the strong effect of orange light in the cyanobacterium *Synechocystis* sp. PCC 6803 are phycobilins. Phycobilins are open tetrapyrrole pigments that are covalently bound to proteins forming large globular moieties, denoted phycobilisomes. Such phycobilisomes are found in cyanobacteria, red algae and glaucocystophytes. Different forms of these water soluble pigments absorb light from the green to red range of wavelengths. Some cyanobacteria may respond to the wavelength distribution by complementary chromatic adaptation in which the constituents of the phycobilisomes change.^30^ Carotenoids build the final group of relevant pigments. They exist in a large group of variations, with over 50 known carotenoids which contribute to light harvesting in photosynthesis, in different species.^31^ Despite their large diversity, the absorption properties of carotenoids actually differ less dramatically than those of chlorophylls or phycobilins, though. Carotenoids absorb primarily blue–green light between 400 and 550 nm, with occasional weak bands in the red region. They are therefore especially important for light‐harvesting in brown algae.^32^

The absorption characteristics generally depend on the environment of the pigment molecule. In protein environment, absorption spectra of the chlorophylls can be shifted by 10–15 nm, often toward longer wavelengths^33^ (an example of this is seen by comparing the positions of the absorption peak attributed to chlorophyll *a* in methanol, and the corresponding peak in pumpkin thylakoids, Figure 1). In addition, the absorption bandwidth is often higher in protein environment than in solution because of the stronger molecule interaction.

The large variation in the spectral responses of photo­synthesis of aquatic organisms (Figure 2) presents an important challenge towards adjustment of the incoming (solar) spectrum. However, it also offers promising and versatile pathways for better harvesting and utilization of sunlight. A good match between the spectrum of the incident light and the absorption spectrum of the photosynthetic organisms is particularly important when the amount of pigments in the illuminated volume is moderate.

## 3 Artificial Photosynthesis

**Figure 3:**
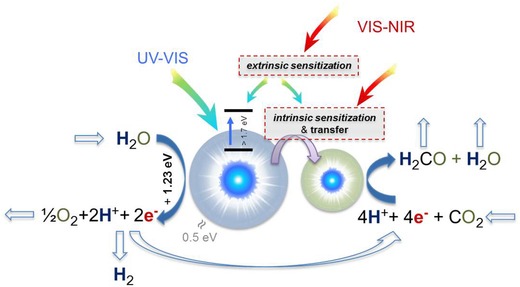
Tandem machinery of an artificial photosynthetic process with intrinsic or extrinsic sensitization.

In nature, oxygenic photosynthesis splits water into oxygen, protons and electrons, which are then used to reduce carbon dioxide and generate carbohydrates. In analogy, artificial photosynthesis (AP) was pioneered by Fujishima and Honda,^34^ initially targeting splitting of water into oxygen and hydrogen at a titanium dioxide semiconductor electrode in a photo‐electrochemical (PEC) cell. Since the original reports, extensive research has been conducted towards enhancing the photocatalytic activity of semiconductor electrodes in PEC systems for a wide range of technical applications, including solar‐driven hydrogen generation, solar fuels, CO_2_ storage, decomposition of pollutants, sustainable biomass production and photo‐synthesis of fine‐chemicals.^35^, ^36^, ^37^, ^38^, ^39^, ^40^ More generally, AP mimics the principles of natural photosynthesis to store incident solar energy in chemical carriers such as hydrogen or carbohydrates.^41^, ^42^ Analogous to its natural counterpart, the AP machinery consists of a light‐harvesting mechanism and a catalytic converter, schematically shown in **Figure** **3**. The minimum energy which the catalyst has to provide for water oxidation and splitting is 1.23 eV.^41^ Due to systematic overpotentials, e.g., induced by reaction kinetics, the experimentally needed value is rather ≈1.7 eV. This sets an initial, simplistic selection rule for potential photocatalytic materials useful in AP: a semiconducting material is required which, upon light absorption, generates an electron–hole pair with sufficient potential energy difference to facilitate the splitting reaction, at experimentally reasonable reaction kinetics. In addition to the most prominent candidate, TiO_2_,^34^ other (transition) metal oxides such as Cu_2_O,^43^ α‐Fe_2_O_3_,^44^ WO_3_,^45^ BiVO_4_,^46^ Sr_1–*x*_NbO_3_,^47^ SnO_2_,^48^ and oxides of indium^49^ or manganese,^50^ metal sulfides, (CdS, ZnS^51^) and chalcopyrites,^52^ or metallic nanoparticles and thin layers such as of ruthenium^53^ have been investigated (for some reviews, see Refs. 54, 55, 56. As a rapidly emerging alternative for these materials, graphitic carbon nitrides (with the presently most stable form of g‐C3N4), have been proposed.^57^, ^58^ Meanwhile, the use of such carbon nitride photocatalysts has been demonstrated in a variety of applications, e.g., water oxidation,^59^ water reduction^60^ and CO_2_ reduction.^61^

Regardless of the catalyst employed, the overall process of photosynthesis (illustrated in Figure 3) requires efficient coupling between light absorption and charge separation in the catalyst, H_2_O oxidation, and the formation of molecular hydrogen, or energy and proton transfer for CO_2_ reduction and the synthesis of carbohydrates.^62^, ^63^ In this complex interplay between absorbers, converters, catalyzers and transducers, photons, electrons and transfer processes operate within a broad range of scales of time, energy and length.^64^ This is presently posing significant limitations on the design of artificial photosynthetic devices for solar‐to‐fuel conversion.^65^ Extrinsic sensitization might provide an alternative approach for this, as it allows for reducing internal system complexity by adjusting the incoming photon spectrum.

As noted above, large‐bandgap semiconductors are generally required for water splitting. For example, the three photo­catalytic polymorphs of TiO_2_, anatase, brookite, and rutile, provide a band‐gap energy of 3.23, 3.14, and 3.02 eV, respectively.^66^, ^67^ These energy values correspond to absorption edges of about 383, 395, and 410 nm. At lower incident photon energy, photo‐induced charge‐separation is not efficiently possible in pure titania. On the other side, the UV spectral range comprises not more than roughly 10% of the incoming solar photon flux at terrestrial air mass,^54^ see also Figure 1. At the same time, the otherwise unused NIR tail of the solar spectrum (at photon energies <1 eV) represents more than half of the total energy flux of the incoming solar irradiation. The region of natural photosynthetic activity comprises little more than a third of the incoming sunlight.^68^ To overcome this apparent mismatch, band‐gap adjustments through anion exchange, for example, in titanium oxy‐nitrides,^69^ through deep reduction such as in black titania, TiO_2–*x*_,^70^ or through the use of alternative semiconductors with lower band‐gaps (see above) is possible to a certain extent. However, in such a case, secondary problems such as photo‐bleaching, thermalization and electron recombination have to be taken into account. Yet another alternative again mimics natural photosynthesis, where intrinsic sensitization is achieved through the use of chromophores (photosensitizers) such as ruthenium complexes with a small band‐gap, which are attached to the semiconductor.^71^, ^72^ The best‐known example of this is the dye‐sensitized solar cell.^6^ It uses a photoactive low‐bandgap dye so as to generate, upon illumination, a photoelectron which is subsequently transferred to the conduction band of a titania photoelectrode. However, in order to set‐up a full photosynthetic process, again, a complex tandem of at least a water‐oxidation catalyst and a hydrogen‐evolution catalyst are required, together with a suitable transducing mechanism (Figure 3). Incomplete spectral harvesting hence remains a pressing issue.

## 4 Solar Spectral Conversion in Photochemistry and Photosynthesis

**Figure 4:**
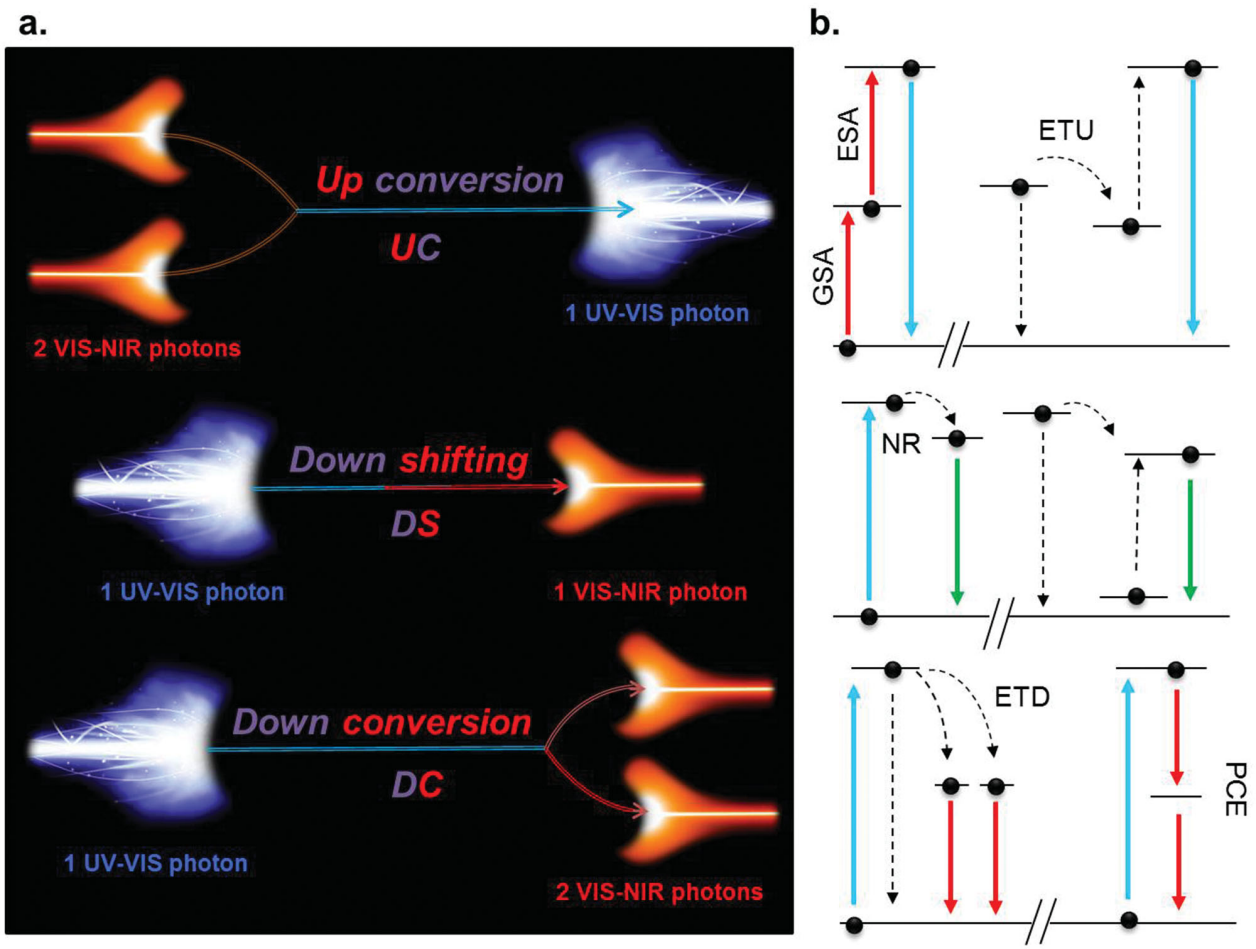
Principles of spectral conversion through photoluminescence. a) In up‐conversion, two low‐energy photons are converted into one high‐energy photon. Down‐conversion and down‐shifting rely on conversion of a high‐energy photon into one or more low‐energy photons. b) A simplistic scheme of the associated electronic transitions in the active center. UC, as a two‐photon process, involves coherence of ground state (GSA) and excited state absorption to generate high‐energy photons by direct relaxation or through energy transfer (ETU). In DC, emitted photons may derive from photon cascade emission (PCE) or from energy transfer reactions (ETD). For clarity, non‐radiative relaxation (NR) is depicted only for the DS reaction.

The use of photoluminescent materials for concentration and spectral modification of solar irradiance has been considered since the 1970s.^73^, ^74^, ^75^ As has already been noted, such solar spectral conversion has widely been employed as a concept for extrinsic PV cell sensitization, and is still receiving notable attention within this area.^3^, ^4^, ^5^ Illustrated schematically in **Figure** **4**, spectral adjustment through photoluminescence typically comprises conversion of a high‐energy photon into one or more low‐energy photons (down‐shifting (DS); down‐conversion (DC),^76^ or of two or more low‐energy photons into one photon of higher energy (up‐conversion (UC).77 All three processes can be used for extrinsic sensitization, as they allow for spectral adjustment of the incoming light. DC (often also denoted *quantum cutting*) additionally increases the number of available photons and may hence—at least theoretically—enhance quantum efficiency to above unity.^78^, ^79^

### 4.1 Up‐Conversion

Spectral blue‐shifting by up‐conversion luminescent materials appears most relevant for sensitization of semiconductors or pigments with a high bandgap, i.e., >1.7 eV. Consequently, up‐conversion has been proposed for sensitizing photocatalysis at TiO_2_, Bi_2_WO_6_, α‐Fe_2_O_3_, CdS, and other semiconductor surfaces^80^, ^81^, ^82^, ^83^, ^84^, ^85^, ^86^, ^87^, ^88^, ^89^, ^90^, ^91^, ^92^ and, more recently, also to increase the photosynthetic activity in pumpkin leaf thylakoids.^93^ and *Chlorella vulgaris* microalgae.^94^

In these studies, a broad variety of combinations of up‐conversion materials with photocatalysts has been described (for a review on photon up‐conversion in photocatalysis, see, e.g., Ref. 95. It is the present understanding that rare‐earth‐ (RE) doped materials represent the most promising candidates to assist long‐wavelength light harvesting of solar irradiation through photon up‐conversion (see also Figure 1a). Among the most prominent inorganic host species are the fluorides of yttrium, in particular, NaYF_4_, for UC as well as for DC.^95^, ^96^, ^97^, ^98^, ^99^ Even though the electronic transitions within the Xe4f configuration of the typical RE^3+^ (lanthanide) activator species are forbidden by quantum mechanical selection rules, crystal‐field‐induced intermixing of the f states with higher electronic configurations yield very long decay times and, consequently, a strongly enhanced probability of sequential excitations and excited state energy transfer, which are both a prerequisite for efficient up‐conversion luminescence.^77^

For example, Qin et al.^92^ and similarly also Ren et al.^86^ reported on near‐infrared photocatalysis using up‐conversion in YF_3_:Yb^3+^–Tm^3+^/TiO_2_ core–shell nanoparticles. Li et al.^81^ explored a novel near infrared photocatalyst by combining low band‐gap CdS with an up‐conversion material, demonstrating NIR photodegradation of Rhodamine B and methylene blue. Wang et al.^87^ investigated the degradation of ethyl violet using visible light and a rutile catalyst in the presence of Er^3+^‐doped (Ba,Cd)F_2_. They subsequently suggested application in (waste) water treatment. In the same context, RE‐doped TiO_2_ nanocrystals were also considered.^84^, ^89^ Other reported options for TiO_2_‐NIR extrinsic sensitization are, e.g., Er^3+^‐doped YAlO_3_ 100, 101 or Y_2_O_3_:Yb^3+^,Tm^3+^.^82^ The NIR sensitization of water splitting at α‐Fe_2_O_3_ through Er^3+^ and Yb^3+^‐doped NaYF_4_ nanoparticles was reported by Zhang et al.^83^ In yet another example, Zhang et al. employed direct doping of photocatalytic Bi_2_WO_6_ with Er^3+^, again extending its range of activity through NIR photon up‐conversion by demonstrating enhanced degradation of Rhodamine B and of phenol.^90^

**Figure 5:**
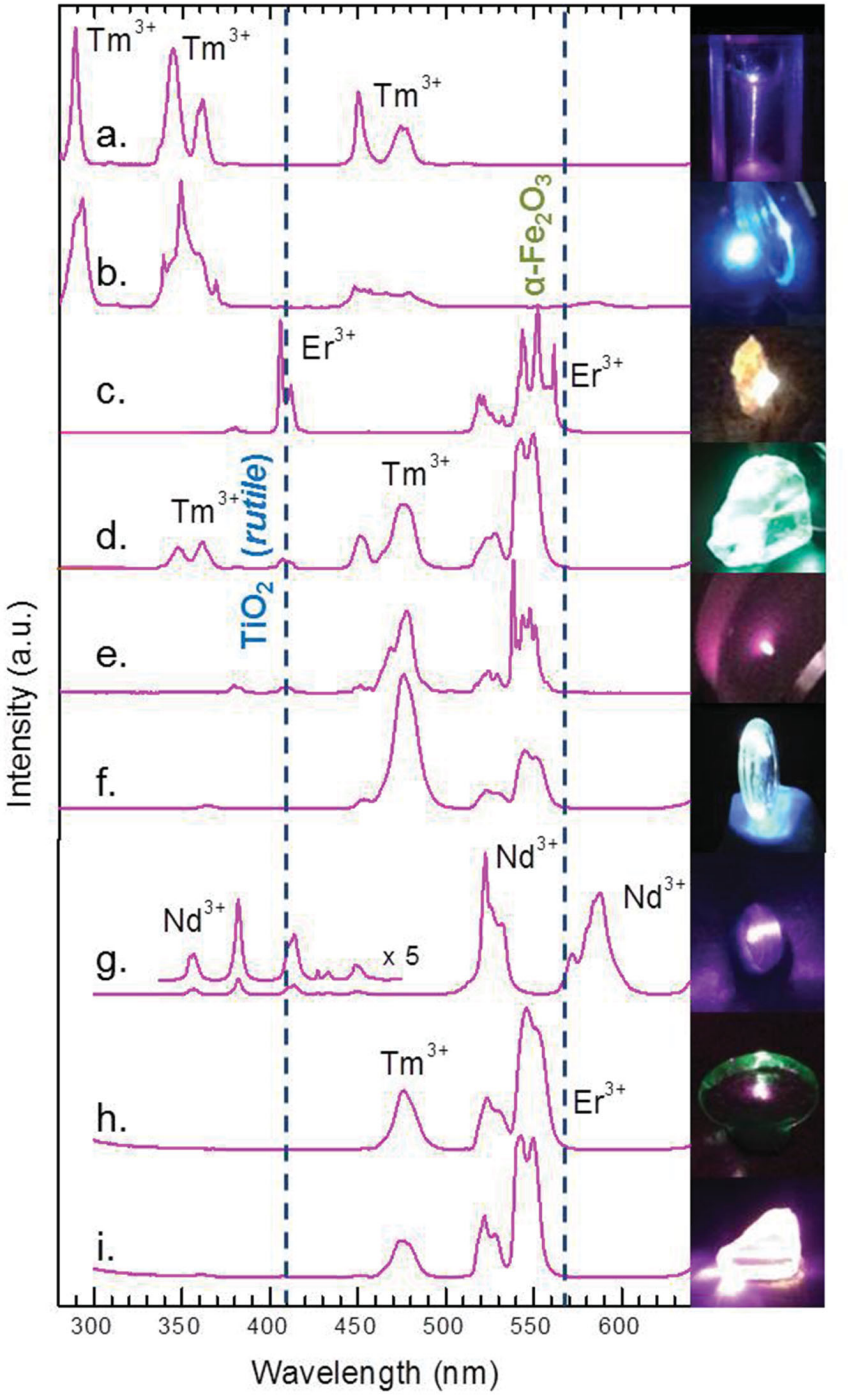
Normalized up‐conversion spectra in the UV–vis range for a variety of RE‐doped luminescent materials: a) NaYF_4_ core‐shell nanoparticles,^104^ b,c) K_2_YF_5_ crystals^105^ d,i) ZBLAN fluoride glasses,^88^, ^102^ e) RE‐doped organic resins,^107^ f,h) oxyfluoride glasses,^103^ and g) nanocrystalline oxyfluoride glass ceramics.^85^ Spectra were taken at excitation with a 980 nm (a–f) or a 800 nm (g–i) laser diode, exciting the Yb^3+^ and/or the Er^3+^ and Nd^3+^ antenna ions. The corresponding sample photographs (right) illustrate the conversion of invisible IR light into visible light.

The overall reaction scheme of all these examples is very similar and rather straightforward. We illustrate this in **Figure** **5**, which summarizes a broad range of (nano‐)crystalline, glassy and glass ceramic up‐conversion materials.^88^, ^102^, ^103^, ^104^, ^105^, ^106^, ^107^ In all these materials, a lanthanide dopant acts as antenna (absorber) for NIR or visible photons (Figure 1) which are converted according to Figure 4. The materials which are exemplarily presented in Figure 5 range from RE‐doped ZBLAN fluoride glasses,^88^, ^102^ oxyfluoride glasses^103^ and glass ceramics,^85^ K_2_YF_5_ crystals,^105^ NaYF_4_ core‐shell nanoparticles,^104^ and RE‐doped organic resins for use in 3D‐printing technology.^107^ The shown emission spectra have been recorded during excitation with laser diodes at 980 nm and at 800 nm, respectively, to efficiently address the exemplary Yb^3+^, Er^3+^, and Nd^3+^ antenna ions (see Figure 1). As exemplary emitting species, Er^3+^, Tm^3+^, and Nd^3+^ are employed. For all emitters, the high‐energy emission bands can be used to bridge the band‐gap of a suitable semiconductor for generation of an electron–hole pair as the first step in the photosynthetic procedure. To illustrate this, the band‐gap energies of TiO_2_ (rutile, 3.02 eV) and α‐Fe_2_O_3_ (2.18 eV) are indicated in Figure 5. For example, addition of ZBLAN fluoride glass (curve (d) in Figure 5) causes an up to 20% improvement of the photocatalytic activity of a benchmark TiO_2_ photocatalyst in the decomposition of methylene blue in water under Xe lamp irradiation has been obtained.^88^

At present, all applications of photon up‐conversion in photo­chemistry have been performed on comparably limited laboratory scale and using mostly model reactions (such as, e.g., the degradation of certain dyes), which do not necessarily represent the anticipated application. On the other hand, spectral up‐conversion, if conducted efficiently, clearly has an unambiguous effect on NIR harvesting in photosynthesis and photocatalysis. On materials scale, today's applications mostly present simplistic proofs of principle. Further exploration of photoluminescent converter materials such as, for example, the consideration of transition‐metal‐doped chromophores^108^, ^109^, ^110^ leaves plenty of room for future improvements (see following section). However, important advances are now especially necessary, not only in the exploration of new and improved materials, but also in the consideration of real‐world demonstration scale, potential system integration, extrinsic efficiency, and life cycle analysis.

### 4.2 Down‐Shifting and Down‐Conversion

In the simplest functioning of photoluminescence, a photon of higher energy is converted to a photon of lower energy (DS, Figure 4). Application of DS for spectral conversion is hence very straightforward. Depending on light scattering at the converter, extrinsic quantum efficiency and secondary absorption effects, DS always results in an increase in the number of available photons at the targeted emission energy. Its potential benefit has now been demonstrated for various natural photosynthetic organisms, including *H. pluvialis* (≈30% and ≈18% increase in reproduction and in oxygen production rates, respectively^7^) and other microalgae species,^111^, ^112^ but also higher plants such as spinach (≈25% increase in CO_2_ assimilation rate^9^). Such benefit, on the other side, relies on the simple fact that UV light in particular, but also other high‐energy parts of the solar spectrum simply do not contribute to photosynthesis because of tissue transparency or reflectivity, or because they trigger photoinhibition.^113^, ^114^ This is usually not the case for photocatalytic materials, especially those having a high photoelectronic band‐gap.

In DS, the energy difference (Stokes‐shift) between incoming photons and emitted photons is lost through non‐radiative relaxation. Efforts to harvest this energetic fraction have led to the concept of quantum cutting and DC (Figure 4), where the Stokes‐shift is high enough to allow for a second (or more) photons to be generated following absorption of a single incoming photon to increase the overall quantum efficiency to beyond unity.^115^ Consequently, DC does not strictly target (extrinsic) sensitization, but multiplication of photons. Due to this intriguing feature, materials which enable efficient DC luminescence have been receiving significant attention for improving solar cell efficiency over the last decade.^76^, ^79^ Most DC materials provide NIR photon generation, for example, using Yb^3+^ as the emitter species and one or more sensitizers for broadband absorption.^116^, ^117^, ^118^ Efficient quantum cutting phosphors for visible light generation are still rare.^119^ For application with natural photosynthesis, DC presently appears less interesting because of simple physical limitations. For example, to generate, through DC, two photons at the peak photosynthetic activity of typical leaf thylakoids (≈680 nm, 1.8 eV), a photon of at least 3.7 eV (<335 nm) would be required in an ideal converter material. Such photons, even when converted very efficiently, represent only a rather small fraction of the solar spectrum. Broadband UV‐ or even green‐to‐red‐conversion, on the other hand, is not possible by 1:2 DC. For artificial photosynthesis, taking into account typical over‐potentials requires bridging an energy of ≈1.7 eV (730 nm), i.e., DC absorption at <365 nm (>3.4 eV). In this case, the action spectrum of today's DC materials could eventually be approached, but a notable reduction of over‐potentials would still be highly desirable.

### 4.3 Efficiency and Benefit of Spectral Conversion

The efficiency of solar‐to‐chemical energy conversion is defined as the ratio between chemical potential energy—for example, in the form of hydrogen—and incoming radiant energy. Here, extrinsic sensitization through spectral conversion acts in two principle ways: for one, the transduced radiant energy is reduced due to the Stokes‐shift of the photoluminescent converter material, and, in the case of UC or DS, also due to its intrinsic quantum efficiency, i.e., the number of emitted photons relative to the number of absorbed photons. This contributes negatively to energy conversion efficiency. The positive contribution is supposed to arise from increasing the number photons in the sensitive spectral region of the photoelectric material. Such an increase can obviously be obtained only when the number of photons which are converted into the target spectral regime is higher than the number of photons which are absorbed from this regime (an aspect which is sometimes disregarded in UC upon broadband illumination with solar light).

As a third, indirect aspect, scattering and reflection have to be taken into account; they may have positive as well as negative effects. Following this consideration, the actual benefit of providing more photons to the photochemical reaction has to be revisited. It clearly differs between natural and artificial photosynthesis.

#### 4.3.1 Photoconversion Efficiency in Natural Photosynthesis

For the case of natural photosynthesis, a simplistic estimate of overall photoconversion efficiency in higher plants has been provided by Zhu et al.^120^ Considering spectral selectivity, surface reflection, the difference between absorbed photon energy and the energy of charge separation, photorespiration, and the efficiency of energy transduction to carbohydrate synthesis, they arrive at a theoretical limit of 4.6–6.0% of solar‐to‐chemical conversion efficiency. Experimental observations are typically well‐below this value. For example, closed‐cycle algae reactors may reach up to about 3% of photoconversion efficiency,^121^ however, at rather high operational cost relative to the more widely employed open ponds.^122^ In the present context, it has to be noted that for given reference conditions, the photo­synthetic yield is dependent on incoming photon flux only at relatively low irradiation intensity, as saturation and photo­protective reactions start already at an incoming radiant flux of approximately 70 μmol m^−2^ s^−1^ (corresponding to roughly 3–4% of the full solar irradiation at air mass (AM) 1.5). Full saturation occurs when no more increase in the photosynthetic reaction rate with increasing irradiance is observed, obviously strongly depending on plant or algal species, but in land plants roughly within 1000–1500 μmol m^−2^ s^−1^, i.e., at 50–70% of the standard AM 1.5 solar irradiance. This aspect has to be critically taken into account when considering the straightforward application of spectral conversion to natural photosynthesis. Clearly, the potential benefit very strongly depends on the actual target application and on the design of the respective system. In particular, in most cases, not the simple provision of more “good” photons, but their spatial and temporal distribution will be of importance. Also, not quantitative, but qualitative, spectral adjustment might be a target application.

#### 4.3.2 Photoconversion Efficiency in Artificial Photosynthesis

The situation is very different in artificial photosynthesis. For example, for water splitting, a target value of 10% of photoconversion efficiency is considered as the break‐even towards larger‐scale commercial implementation.^123^ In particular, such a value beats the combination of a photovoltaic harvesting scheme and a commercial electrolyzer.^124^ The present record on laboratory scale has been reported at 12.3% using a perovskite solar cell and a NiFe layered double hydroxide catalyst.^125^ Other than in natural photosynthesis, here, the number of photons which are provided to the photochemical machinery has a dominating effect on the overall yield even at elevated irradiation intensity. The major limiting factors are, besides the shape of the incoming spectrum, the bandgap of the semiconductor (or dye) species, the occurrence of over‐potentials and the shift between photon energy and energy of charge separation. Spectral adjustment through photoluminescence, hence, directly acts on overall efficiency. Momentarily disregarding system cost and complexity, it therefore appears straightforwardly beneficial.

#### 4.3.3 Benefit of Spectral Conversion in Photosynthesis

DS, DC and UC all aim at increasing the number of photons within a specific wavelength regime. With the fundamental prerequisite that the number of available photons is actually a limiting factor in the solar‐to‐chemical energy conversion system of interest, estimating the potential gain in overall efficiency still remains a challenging task. As noted in the previous paragraph, DC presently appears not efficiently applicable, per se, to natural photosynthesis with solar illumination for simple energetic reasons. The potential benefit of DS, on the other hand, is most readily understood for natural photosynthesis, where it is a direct result of spectral adjustment through a simple single‐photon process. As long as the photosynthetic machinery is not saturated and provided that only inactive photons are absorbed by the converter, it solely depends on absorption cross‐section, internal quantum efficiency and remission efficiency of the converter. The energetic efficiency of a DS process is further limited by quantum efficiency and by the Stokes‐shift, which is about 0.6 eV between green and red (accounting for an energetic loss of roughly 25% per converted photon). Further considering green‐to‐red conversion, the green part of the solar spectrum represents about 9% of the radiant energy. If fully converted and assuming a photosynthetic quantum efficiency of 100% and the absence of any saturation effects, this lets expect a maximum increase photosynthetic turnover of 40–50%. This estimate is close to the observed experimental improvements which have been reported for the application of such spectral conversion in natural aquatic photosynthesis.^7^

There is more controversy in the consideration of UC. UC is, per se, a multi‐photon process. Its efficiency therefore largely depends on the absorption cross section, coherence and lifetimes of the participating excited states (Figure 4), and can strictly be improved only through increasing the number of excitation photons and/or increasing the lifetime of the intermediate excited state. In general, the accurate determination of the absolute quantum efficiency of UC processes remains a disputed issue. Probably for this reason, many reviews of potential materials (e.g., Ref. 86) simply disregard any critical discussion or calculation of up‐conversion quantum yield (and sometimes jump over the fact that not sunlight, but a laser was used for excitation in most cases). A quantitative method has been proposed by Suyer et al.^126^ Within their formalism, the intensity of the up‐conversion emission is characterized in terms of a photon flux calculation on the spectra through a comparative band area evaluation so as to determine the number of up‐converted photons relative to the total number of NIR excitation photons absorbed by the material. This relies on very careful, quantitative spectroscopy where the actual photon flux is determined on excitation as well as on emission spectra. For example, for the data shown in Figure 5, the number of up‐converted photons with respect to the total number of photons emitted from the sample is ≈46% and ≈66%, respectively, for the ZBLAN glass (curve (d)) and for the K_2_YF_5_ crystal (curve (b)). Such values, however, have only limited relevance in the determination of the quantitative benefit of UC in an energy conversion process. A more helpful simplistic construct of up‐conversion yield has been proposed where the emitted light power is related to the absorbed light power, taking into account that emission and (self‐) absorption occur in parallel within the target spectral ranges of excitation and emission. Then, UC yield is a product of quantum efficiency of the actual UC reaction (determined from, e.g., actual and 0 K lifetime of the relevant excited state), the emission intensity of up‐converted light relative to the intensity of direct emission (from the intermediate level, Figure 4b), and the relative absorption power in the visible (target emission) spectral regime versus that of the IR (excitation) regime.^127^ For state‐of‐the‐art materials, such a calculation leads yield values of ≈0.5%.^128^

That said, despite notable progress in recent years where UC schemes are now available which operate also under normal or concentrated solar illumination (in contrast to model studies which rely on laser excitation), further development is still necessary to achieve a breakthrough which will allow for actual real‐world applications.^5^

## 5 Concepts and Demonstrators

Today's consideration of (solar) spectral conversion with energy harvesting needs to overcome the asymmetric focus on novel materials in favor of system design, exploitation and implementation with conceptual demonstrators. Those should showcase concrete versus claimed benefit (and advantages over alternative routes such as intrinsic sensitization or artificial lighting). Benefit may thereby arise not only through the quantitative promise of increased absolute efficiency, but also on less obvious aspects such as qualitatively improved or tailored spectral match with specific absorption characteristics (e.g., for super‐selective synthesis which, at present, requires artificial illumination), improved areal and temporal energy distribution, or improved spatial and temporal coherence. While a variety of demonstration reports have been mentioned in the previous paragraphs, implementation beyond laboratory scale or even commercial exploitation still requires intense efforts of research and development.

**Figure 6:**
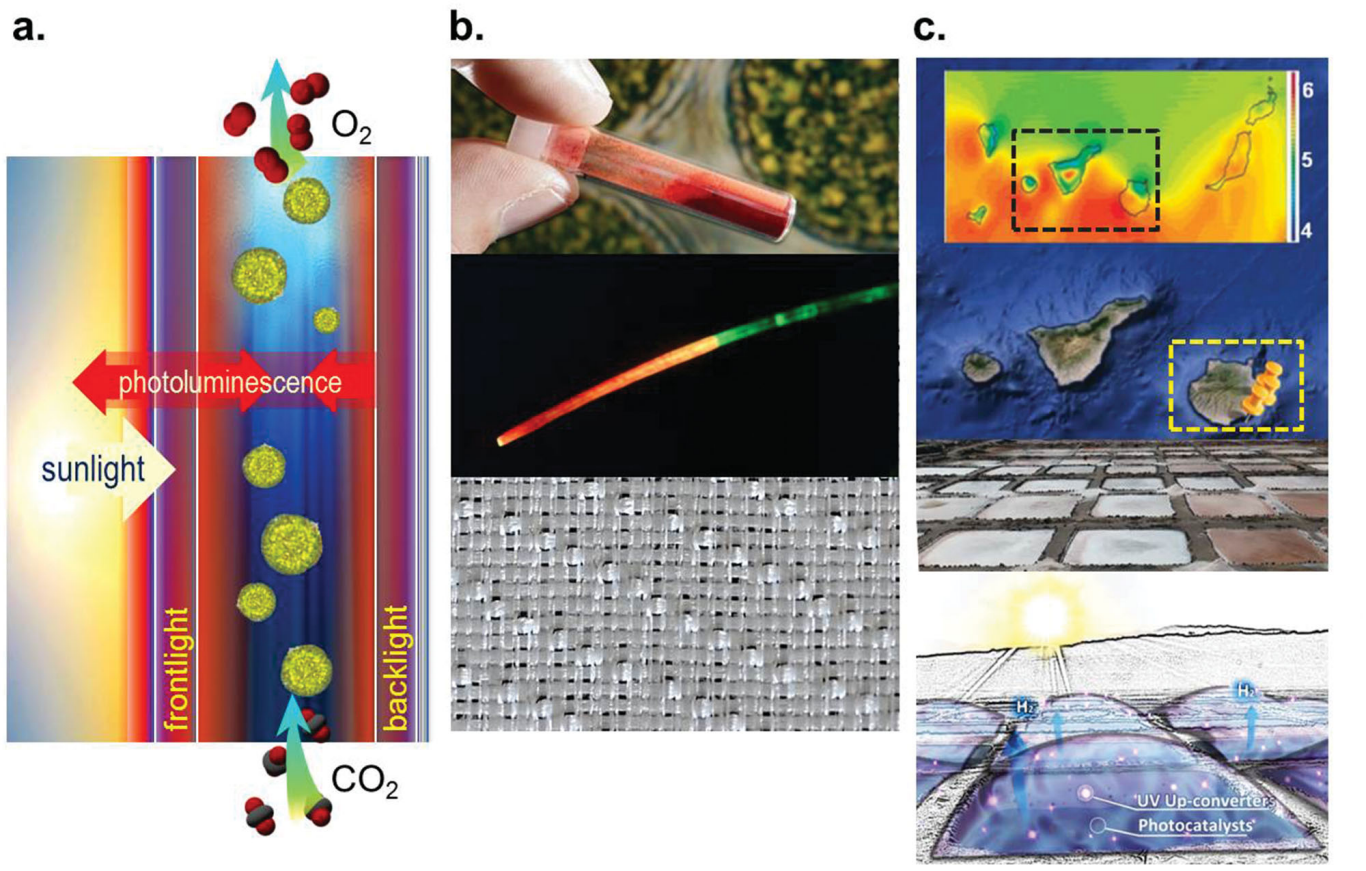
Conceptual approaches for implementing extrinsic spectral conversion with natural and artificial photosynthesis. a) The design of a frontlight (left) and a backlight (right) photoluminescent converter and its application with a flat‐panel microalgae reactor.^7^ b) A typical phosphor material (here: (Ca,Sr)S:Eu^2+^ for green‐to‐red downshifting (top). This phosphor can be coated on a light‐guiding and light‐concentrating optical fiber—example shown here (middle): 0.5 mm in diameter, PMMA, front‐end (left) coated with (Ca,Sr)S:Eu^2+^—for tailored light delivery. Large‐area fabrics can be manufactured from such fibers (bottom, showing un‐coated fiber). c) Summary of an exemplary concept of seawater‐splitting, where the intense solar irradiance of the Canary Islands (top, in kWh m^−2^ per day) and the existing infrastructure of salt‐flats (middle) are used as the basis for H_2_ generation in shallow, covered ponds which comprise slurries of photoconverters and photocatalysts in a combination of back‐ and frontlight converters. Reproduced with permission.^88^ Copyright 2013, Royal Society of Chemistry.

Present concepts for extrinsic sensitization of photosynthesis reach from specialty flow‐through (flat‐panel) reactors^7^ to greenhouses.^8^, ^9^ Noteworthy, many such agricultural or aquaponic applications rely on artificial lighting (e.g., Refs. 129, 130) In the three mentioned studies, the objective was therefore not alone the advertised efficiency increase in solar harvesting, but also the replacement of artificial light sources by solar‐activated phosphors so as to reduce overall energy consumption. The employed phosphor materials strongly resemble those which are used in, e.g., fluorescent lamps or WLED systems, only that they are tailored in terms of their spectral properties to better meet the specific requirements of photosynthesis and excitation through sunlight (versus artificial UV or blue irradiation). Also the foremost criteria for each phosphor's suitability resemble those which apply to phosphors in lighting, i.e., a quantum efficiency which exceeds 90%, and very high emission stability under long‐term continuous or cycling excitation and thermal load. DS has consequently been in the focus of the above‐noted applications to natural photosynthesis. For example, using long‐lasting DS phosphorescence from Eu^2+^/Cu^+^‐co‐doped (Ca,Sr)S, Lian et al. proposed implementation with a large‐area plastic film into greenhouse roofings.^8^ Following two field tests, they reported an efficiency gain in terms of crop yield of about 21.3% and 23.9%, respectively, however, without giving details on specific experimental conditions. Such a roofing situation presents a typical front‐light design where incoming light passes through a semitransparent sheet of material comprising the photoluminescent converter material (or activator ions, e.g. Ref. 131). It is schematically depicted in **Figure** **6**a. Since photoluminescent emission scatters in all directions, this requires a further scheme for avoiding remission of the just‐converted photons back into the outside atmosphere. The latter issue is avoided in the backlight‐design, where the light first passes the reactor and only the remaining, transmitted fraction falls onto the photoluminescent converter, from where it is remitted into the reactor (Figure 6a, right).

Another situation which does not primarily target a simple increase of a certain fraction of photons is light delivery. Here again, natural photosynthesis and, in particular, aquatic photosynthesis are primarily addressed. As has already been noted, in both cases, solar energy harvesting occurs at the complex trade‐off between rapid oversaturation, shading and inefficient areal distribution of light. Effective delivery of, not the maximum, but the optimum photon flux to as many photoreceptors as possible with as little as possible temporal and spatial fluctuations is the key interest. For example, in a flow‐through microalgae reactor, light penetration is limited to only a few millimeters, depending on algae load which directly determines optical density. Therefore, high throughput in terms of number density of algae either reduces the light harvesting efficiency, or requires thinner reactor cavities (which again reduces throughput). In a somewhat contradictory compromise, an intermediate solution to this problem has been proposed where the light‐harvesting capacity of the algae is reduced, for example, through antenna size reduction, so as to enable deeper light penetration.^132^, ^133^, ^134^ However, regardless of its practical applicability, this approach does not, per se, lead to a net increase in system throughput and does not, for example, overcome the issue of shading and light scattering in deep reactors. The most intense efforts to handle the problem of light delivery are presently made in reactor engineering and, for example, through tailoring the flow patterns in aquatic reactors (for example, to constantly mix the system so as to regularly expose any one fraction of the photosynthetic biomass to the illuminated surface). On the other side, volumetric light delivery and solar spectral conversion may present elegant but presently unexploited alternatives. For example, following concepts of solar power concentration (CSP), sunlight can be collected and, through light‐guiding multimode fiber, be transported into a reaction volume. Such fiber can be coated with a photoluminescent spectral converter (Figure 6b) so as to gradually release and, at the same time, convert the collected sunlight. Figure 6b shows such a scenario, using a PMMA fiber in which, in this present example, artificial green light is transported and converted, at the fiber end, into red light for delivery to a photosynthetic reaction. If woven into a (flexible) textile mesh (bottom of Figure 6b) or bundle, volumetric and highly proximal light delivery can be achieved.

Flow‐through photoelectrical reactors which comprise NIR up‐conversion have previously been proposed conceptually, Figure 6c.^88^ It was suggested to exploit shallow water beds for solar‐driven seawater splitting,^135^ using the already existing infrastructure of traditional salt flats, e.g., on the Canary Islands, where high levels of solar irradiation (up to 6.0 kWh per m^2^ per day) are available throughout the year,^88^ notably well in the considered range of the US road map analyses and report on PEC hydrogen production.^136^ As has previously been pointed‐out,^136^, ^137^ arrays of slurry‐type seawater pools with a depth of a few cm could thus be operated as solar‐to‐fuel generation plants. Such arrays and aqueous trough systems would be covered by overlying plastic films to retain the electrolyte and synthetic gases while enabling light penetration. Thereby, the photo­luminescent converter material may be both compounded into the plastic film (frontlight converter) and dispersed within the pool reactor (backlight converter). Here, a pressing issue remains the output of synthetic gases per illuminated area of reaction, with a trade‐off similar to the one of photosynthesis: due to the limit of penetration depth (here, of UV light in aqueous suspensions), only shallow reactors are employed. Even when fully exploiting multiple scattering in slurry reactors, the reactive (illuminated) area of (artificial) photosynthesis is low in view of the needs of large‐scale implementation. Therefore, here too, the functioning of spectral converters might be their contribution to more efficient light delivery just as well as the sometimes overly simplified increase of light intensity in a specific spectral range.

## 6 Outlook

Efficient harvesting of solar energy remains a challenging task and, eventually, the only solution to the major problem of ultimately sustainable energy provision. In this context, photochemical energy harvesting and storage through artificial photosynthesis and solar‐to‐fuel approaches remain at a comparably early stage of development, where the approximate break‐even of energetic efficiency at >10% has still to be overcome beyond the laboratory scale. Extrinsic sensitization of photocatalysts through photoluminescent spectral converters may contribute towards this goal, but also requires dedicated design strategies on system scale. Photon up‐conversion appears the most suitable scheme, in this context, to enable harvesting of NIR and visible light with high‐bandgap semiconductors. For down‐shifting, we see primary interest in natural photosynthesis, where qualitative spectral adjustment is required beyond the simple provision of higher photon flux. While physically intriguing, only little interest is momentarily seen in quantum‐cutting due to the fact that the energy window within which spectral tuning is required is relatively narrow, and the excess energy even in the UV tail of the solar spectrum is not high enough to produce a significant amount of photon pairs in the target energy range.

Contrary to application in PV energy conversion, implementation of solar spectral conversion for extrinsic sensitization of natural or artificial photosynthetic machinery is very straightforward. In comparison to intrinsic sensitization, we see less‐strict limitations with regard to quantum coherence. Hence, the approach provides broader flexibility in the combination of active dyes, semiconductors and energy transduction. Similar to spectral converters in PV, we see further interest in the exploration of new materials with improved spectral properties, in particular, for assessing specific combinations of spectral harvesting and remission regimes, and for providing high quantum efficiency in the photoluminescent conversion process. However, systems integration, innovative approaches for light delivery and reactor design represent predominant issues. Subsequently, real‐world demonstrators and life cycle assessment on system scale remain open tasks.
